# Blood-Based Biomarkers for Predictive Diagnosis of Cognitive Impairment in a Pakistani Population

**DOI:** 10.3389/fnagi.2020.00223

**Published:** 2020-07-22

**Authors:** Ghazala Iqbal, Nady Braidy, Touqeer Ahmed

**Affiliations:** ^1^Neurobiology Laboratory, Department of Healthcare Biotechnology, Atta-ur-Rahman School of Applied Biosciences, National University of Sciences & Technology (NUST), Islamabad, Pakistan; ^2^Centre for Healthy Ageing, School of Psychiatry, Faculty of Medicine, University of New South Wales, Sydney, NSW, Australia

**Keywords:** biomarkers, oxidative stress, cognitive impairment, amyloid-beta, serum, tau, metals, lipids

## Abstract

Numerous studies have identified an association between age-related cognitive impairment (CI) and oxidative damage, accumulation of metals, amyloid levels, tau, and deranged lipid profile. There is a concerted effort to establish the reliability of these blood-based biomarkers for predictive diagnosis of CI and its progression. We assessed the serum levels of high-density lipoprotein (HDL) cholesterol, low-density lipoprotein (LDL) cholesterol, triglycerides, total cholesterol, selected metals (Cu, Al, Zn, Pb, Mn, Cad), and total-tau and amyloid beta-42 protein in mild (*n* = 71), moderate (*n* = 86) and severe (*n* = 25) cognitively impaired patients and compared them with age-matched healthy controls (*n* = 90) from Pakistan. We found that a decrease in HDL cholesterol (correlation coefficient *r* = 0.467) and amyloid beta-42 (*r* = 0.451) were associated with increased severity of CI. On the other hand, an increase in cholesterol ratio (*r* = −0.562), LDL cholesterol (*r* = −0.428), triglycerides, and total-tau (*r* = −0.443) were associated with increased severity of CI. Increases in cholesterol ratio showed the strongest association and correlated with increases in tau concentration (*r* = 0.368), and increased triglycerides were associated with decreased amyloid beta-42 (*r* = −0.345). Increased Cu levels showed the strongest association with tau increase and increased Zn and Pb levels showed the strongest association with reduced amyloid beta-42 levels. Receiver Operating Characteristic (ROC) showed the cutoff values of blood metals (Al, Pb, Cu, Cad, Zn, and Mn), total-tau, and amyloid beta-42 with sensitivity and specificity. Our data show for the first time that blood lipids, metals (particularly Cu, Zn, Pb, and Al), serum amyloid-beta-42/tau proteins modulate each other’s levels and can be collectively used as a predictive marker for CI.

## Introduction

Cognitive impairment (CI) is characterized by short and long term memory loss, deterioration of language, inability to execute motor functions, and/or poor recognition of objects, and impairments in executive abilities (American Psychiatric Association, 1996). Timely diagnosis of CI in old age is very important because CI is likely to progress to the development of dementias (Ritchie et al., 2014, 2017; Tang et al., 2020). Improved diagnoses of CI can help in better management of the disease and might delay dementias in these subjects. CI is the major component of the aging process and is associated with a variety of risk factors, such as smoking, high drug, and alcohol consumption, fewer years of formal education, low-income status, various metabolic diseases like hypertension and diabetes (Freedman et al., 2001; Raz and Rodrigue, 2006), elevated levels of metals (Iqbal et al., 2018), brain shrinkage with age (Raz et al., 2004), genetic factors such as APOE copies (Deary et al., 2009) and oxidative stress (Raz and Daugherty, 2018; Gandhi and Abramov, 2012).

Reliable biomarkers for prediction of early CI are needed, although there has been little success in this aspect. Here, we investigated the role of blood-based lipids, amyloid beta-42, tau and metals, and their correlations as potential biomarkers for improved diagnosis of CI. Lipids play an important role in neurodegeneration by promoting oxidative stress and influencing amyloid plaques formation, thus playing a contributory role in CI (Bodovitz and Klein, 1996; Xicoy et al., 2019). Higher blood cholesterol level leads to lipid peroxidation and reduced levels of several antioxidant enzymes (including superoxide dismutase) have been reported (Sparks et al., 1994). Lipids have also been recently used to distinguish between elderly subjects with small vessel disease and “healthy” age-matched controls (Liu et al., 2020). The association of low-density lipoprotein (LDL) cholesterol, high-density lipoprotein (HDL) cholesterol and total cholesterol with cognitive function has been previously reported but remains inconclusive (Postiglione et al., 1989; Wieringa et al., 1997) due to limited availability of data and varied techniques of assessment of lipid profiles (Anstey et al., 2008; Segatto et al., 2014; Zanchetti et al., 2014). It has also been well established that redox-active metals can disturb the equilibrium between free radical generation and antioxidant potential of cells by increasing the levels of reactive oxygen species (Singh et al., 2019).

Amyloid beta-42 as a biomarker in cerebrospinal fluid (CSF) and/or plasma has been extensively studied in Alzheimer’s disease (AD; Vos et al., 2013) and other neurocognitive disorders (Ritchie et al., 2014, 2017). Amyloid beta-42 is a proteolytic fragment of amyloid precursor protein (APP), which is selectively cleaved by beta and gamma-secretase enzymes to yield the toxic amyloid beta-42 peptide (Selkoe, 1994) and its fibrils damage synapses (Chen et al., 2017) and causes oxidative stress (Ferrera et al., 2008). There is contradictory evidence on whether the levels of amyloid-beta in the CSF and/or plasma are clinically relevant biomarkers in AD, since published results are difficult to interpret, and there are variations in study design and methodologies used. For instance, some studies have reported increased levels of amyloid-beta (Vos et al., 2013; Petersen and O’Bryant, 2019; Tang et al., 2020) while others have reported lower levels (Song et al., 2011; Ruiz et al., 2013; Mizoi et al., 2014). However, both of these above-mentioned types of studies concluded that amyloid-beta cannot be an accurate biomarker, suggesting that levels of amyloid beta are certainly modulated by other factors (possibly metals, lipids, and tau) and further studies are required.

Tau proteins are abundant in the central nervous system (CNS) and are mainly active in the distal portion of axons hence stabilizing microtubules (Billingsley and Kincaid, 1997). As compared to the amyloid-beta, tau seems to be a reliable marker. Several studies have reported that elevated levels of total-tau and phosphorylated tau in the CSF correlate well with CI and progressing to dementias (Hansson et al., 2006; Diniz et al., 2008; Mattsson et al., 2009; Monge-Argilés et al., 2010; Olsson et al., 2016). As CI is associated with several neurological disorders establishing selective and specific blood-based biomarkers is challenging (Grundke-Iqbal et al., 1986).

Blood-based biomarkers for CI offer ease of access, and in some cases, plasma markers (plasma tau for AD particularly) show better correlation (Zetterberg et al., 2013). CSF based biomarkers can be reliable, however, CSF acquisition is invasive and difficult to obtain, and sometimes data has to be supported by neuro-imaging scans (Jia et al., 2019), which are expensive and time-consuming (Mapstone et al., 2014). A large amount of CSF is absorbed in the blood, suggesting that proteins present in the serum have the potential to act as biomarkers in the neurodegenerative diseases. Therefore, proteins associated with the axonal injury and damage of the blood-brain barrier may be measured in the serum (Zetterberg et al., 2013). Keeping in view all these factors, we studied selected plasma/sera biomarkers for CI in Pakistani health. The key goal of this study was to observe the association between total and fractional cholesterol with the extent of CI and its correlation with amyloid beta-42 and total tau. Furthermore, we estimated the association between serum total-tau and amyloid beta-42 with various metals in the blood and the stages of cognitively impaired subjects and compared the data with age matched healthy controls. Finally receiver operating characteristic (ROC) analysis was performed to evaluate the specificity and sensitivity of amyloid beta-42, tau protein, and various metals as potential biomarkers for diagnosis of mild, moderate, and severe CI.

## Materials and Methods

### Ethics

This study protocol was approved by the Internal Review Board (IRB) of the Atta-ur-Rahman School of Applied Biosciences, National University of Sciences and Technology (approval letter number 35IRB). The procedures were performed following the code of ethics of the World Medical Association (Declaration of Helsinki). Written informed consent was obtained from all subjects and/or caregivers included in the study.

### Subjects

**Figure 1:**
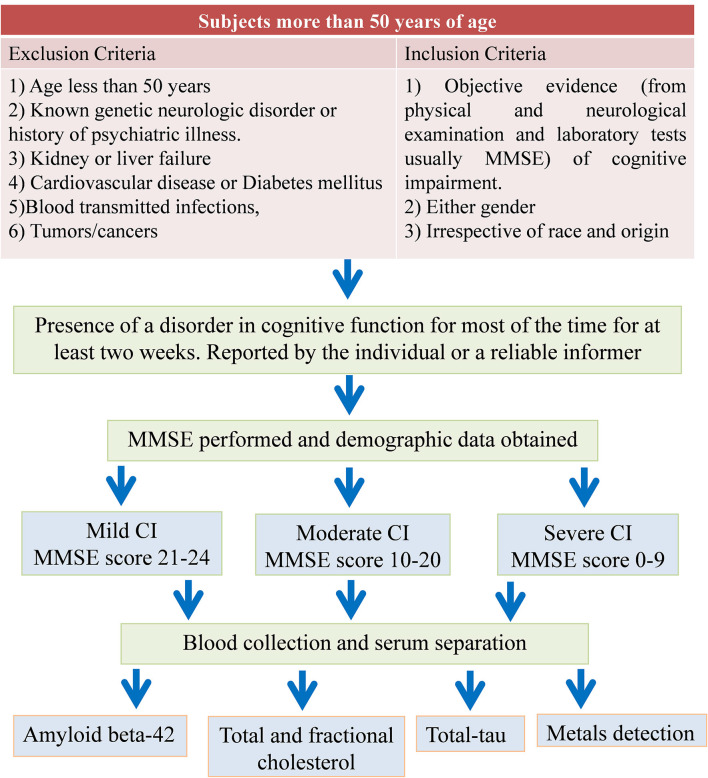
Flow diagram of study design and patients exclusion criteria. MMSE, mini-mental state examination; CI, cognitive impairment.

A total of 273 subjects aged more than 50 years were included in this study. A well-defined inclusion criterion was practiced. Subjects having proper evidence of the CI as reported by neuro physicians or psychiatrists and with more than 50 years of age were included. The patients under any kind of therapies (including the lipid-lowering medications) were excluded from the study. Detailed information was obtained from all subjects through an approved questionnaire. The questionnaire includes the date of birth, occupation, medical history, medications, and lifestyle of the subjects. The study design and the patient’s exclusion criteria are shown in Figure 1.

### Cognitive Function Assessment and Classification of Subjects

Screening of cognitive function was performed using a standard and widely used and accepted 30 point questionnaire test known as the Folstein test or mini-mental state examination (MMSE; Folstein et al., 1975; Ferrante et al., 2019; Nardes et al., 2020). The MMSE was administered by an independent health care provider to evaluate overall cognitive function. The subjects were classified based on MMSE score into mild, moderate, and severe CI (Table 1). In this study of 273 subjects, 72 patients with mild CI, 86 with moderate CI, 25 with severe CI and 90 age-matched healthy control were examined for lipids profiling, total-tau and amyloid beta-42 in serum and correlation of these biomarkers with metals (Al, Pb, Zn, Cu, Cd, and Mn) concentrations were studied. Metals levels in these subjects are reported earlier (Iqbal et al., 2018). All cognitively impaired subjects with co-morbidities were excluded from the study. Older “healthy” individuals aged 50 years, with overall good health and with normal MMSE score were used as age-matched healthy controls in this study.

**Table 1:** Demographic data of the study subjects.

Variables/parameters measured		Age matched healthy control (*n* = 90)	Mild cognitive impairment (*n* = 71)	Moderate cognitive impairment (*n* = 86)	Severe cognitive impairment (*n* = 25)
MMSE score		28.67 ± 1.3	22.7 ± 1	15.7 ± 2.2	5.96 ± 2
Gender	Male	48	33	51	10
	Female	42	39	35	15
Age years (SD)		58.1 ± 6.6	61.7 ± 9	65.1 ± 10.9	79.68 ± 7
Weight kg (SD)		73.07 ± 7.5	71.25 ± 7.2	72.2 ± 7	74.7 ± 5.1
Living standard	Rural	76	59	74	18
	Urban	14	12	12	7
Education	Primary (0–5 yeras)	26	34	43	17
	Secondary level	49	32	39	8
	Higher secondary level	3	3	3	0
	Bachelors and above	12	2	1	0
Marital status	Married	86	64	74	19
	Unmarried	4	8	12	6
Smoking	Smokers	11	21	39	10
	No smokers	89	50	47	15

### Serum Collection

All the procedure was performed under aseptic conditions. Five milliliters of blood was collected by vein puncture from each subject using a sterile needle. Two milliliters was transferred to EDTA vacutainer tubes for metal detection while three milliliters of blood in the other vacutainer was left to clot at room temperature for about 20 min. After that, vacutainers were centrifuged at 3,000 rpm for 10 min to separate the serum from clotted blood. This serum was then immediately transferred to labeled Eppendorf tubes and stored at −80°C for further use.

### Lipid Profile Measurements

Triglycerides, HDL cholesterol, and total cholesterol were measured from serum by Merck Microlab 300 apparatus. Triglycerides were measured by the Abcam triglyceride kit (Catalogue Number: ab65336), total cholesterol was estimated by Merck kit (Catalogue Number: 428901) and cholesterol was calculated by Abcam human ELISA kit (Catalogue number: ab12561). All procedures were carried out according to the manufacturer’s instructions. LDL cholesterol was estimated using the Friedenwald equation i.e.,

LDL cholesterol=total cholesterol−HDL cholesterol—(triglycerides/2.2)

Cholesterol ratio was calculated by the formula

Cholesterol ratio = Total cholesterol/HDL cholesterol

### Total-Tau Level Measurement

Serum Total-tau was estimated using the human tau ELISA kit (catalog number ab210972 and lot: GR302657-1) purchased from Abcam, Cambridge, UK. All the procedures were performed according to the manufacturer’s instructions.

### Amyloid Beta-42 Level Measurement

Measurement of serum amyloid beta-42 was carried out using human amyloid beta-42 ELISA kit (E-El-H0543; lot: AK0017JAN11029) purchased from Elabscience (Hubei, China). The assay was performed according to the manufacturer’s instructions.

### Quantification of Metal Levels and Their ROC Analyses

Blood was digested by a microwave-assisted acid digestion method as previously described (Iqbal et al., 2018). Heavy metals were analyzed in digested blood samples by double-beam Perkin Elmer atomic absorption spectrometer model 700 (Perkin Elmer, USA). Metals levels are reported earlier (Iqbal et al., 2018) and their ROC was analyzed here to find out their diagnostic significance.

### Statistical Analysis

The data were statistically analyzed using GraphPad prism software and variables were compared using one-way ANOVA followed by Bonferroni *post hoc* test. Data were expressed as mean ± standard error of the mean (SEM) and considered significant only if the *p*-value was less than 0.05. Correlation analysis among the two variables was calculated using Pearson’s correlation by GraphPad prism. ROC analyses were carried out to find the cutoff concentration of total-tau, amyloid beta-42, Cu, Al, Zn, Pb, Mn, and Cd.

## Results

### Demographic Data of Subjects

The demographic data obtained from all subject groups i.e., age-matched healthy control, mild CI, moderate CI, and severe CI patients are summarized in Table 1. There were more females compared to the number of males in diseased groups. Most of the subjects were from urban areas. There was less number of individuals with higher education in the case of moderate and severe CI groups. An increasing trend in percent of smokers from mild to severe cognitively impaired groups was observed.

#### Lipid Profile Measurement in Blood Samples of CI Subjects

**Figure 2:**
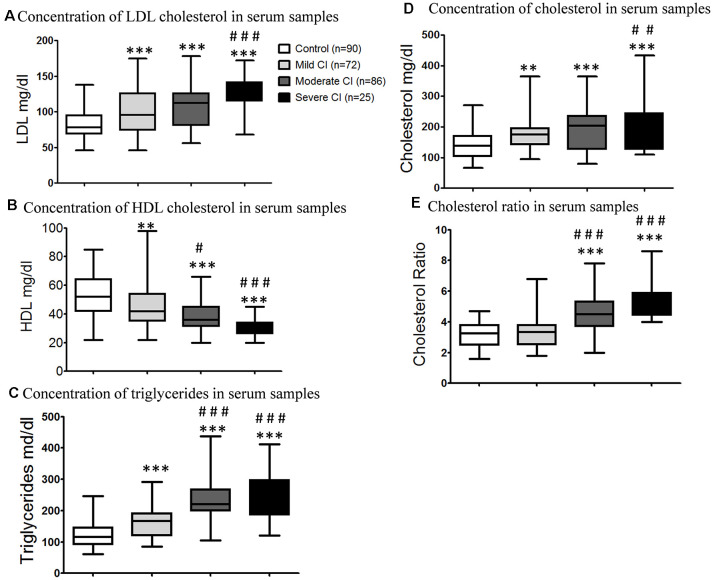
Bar graph showing the total and fractional cholesterol in serum samples of age-matched healthy control, mild CI, moderate CI and severe cognitively impaired subjects. **(A)** Low-density lipoprotein (LDL) cholesterol in serum samples. **(B)** High-density lipoprotein (HDL) cholesterol level in serum samples. **(C)** Triglycerides level in serum samples. **(D)** Total cholesterol concentration in serum samples. **(E)** Cholesterol ratio in serum samples. **p < 0.01, ***p < 0.001 compared with age-matched healthy control group; ^#^*p* < 0.05, ^##^*p* < 0.01, ^###^*p* < 0.001 comparison with mild cognitive impaired group; n, samples size.

The LDL cholesterol concentrations in mild CI (100.5 ± 3.67 mg/dl), moderate CI (107.8 ± 3.01 mg/dl) and severe CI groups (125.8 ± 5.22 mg/dl) were significantly (***p < 0.001) higher than the age matched healthy control group (Figure 2A). The level of HDL cholesterol in serum samples of age matched healthy controls (51.9 ± 1.41 mg/dl) were significantly higher than mild (44.56 ± 1.67 mg/dl; **p < 0.01), moderate (38.26 ± 1.31 mg/dl; ***p < 0.001) and severe (30.68 ± 1.28 mg/dl; ***p < 0.001) cognitively impaired subjects (Figure 2B). It was found that triglycerides levels in serum were significantly higher in mild CI (161.5 ± 5.65 mg/dl; **p < 0.01), moderate CI (230.3 ± 8.1 mg/dl; ***p < 0.001) and severe CI groups (248.5 ± 15 mg/dl; ***p < 0.001) comparative to age matched healthy controls (30.68 ± 1.28 mg/dl; Figure 2C). The total cholesterol levels were significantly lower in age matched healthy control group (138.9 ± 4.49 mg/dl) compared to those diagnosed with mild CI (172.5 ± 5.79 mg/dl; ***p < 0.01), moderate CI (195.8 ± 6.73 mg/dl; ***p < 0.001) and severe CI (217.1 ± 17.48 mg/dl; ***p < 0.001). However there was no significant difference observed among mild CI and moderate CI group and also among moderate CI and severe CI groups (Figure 2D). Cholesterol ratio was significant (***p < 0.001) increased in subjects diagnosed with moderate CI (4.51 ± 0.13 mg/dl) and severe CI (5.28 ± 0.24 mg/dl) when compared to age matched healthy controls. Moreover there was no significant difference among subjects with mild CI (3.43 ± 0.13 mg/dl) and age matched healthy controls (Figure 2E).

#### Correlation Between Lipids Profile and MMSE

**Figure 3:**
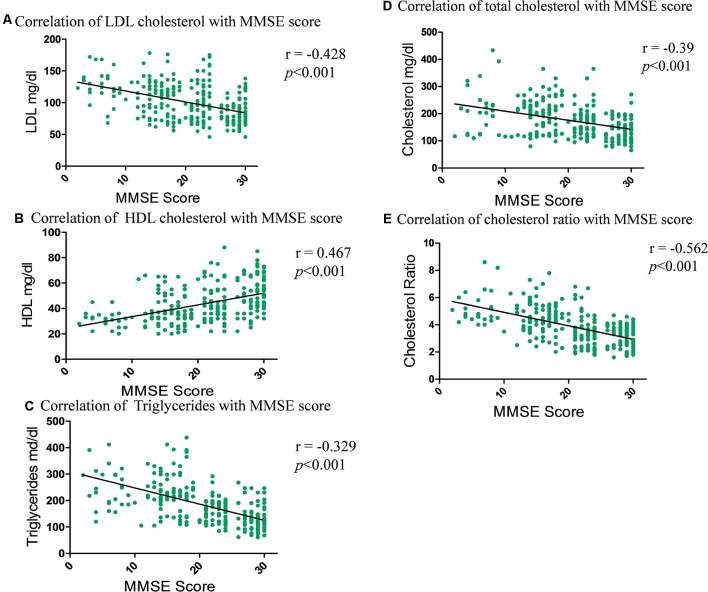
Correlation graph showing the relationship between the total and fractional cholesterol in serum samples and subjects MMSE score. **(A)** Correlation of LDL cholesterol with MMSE scores. **(B)** Correlation of HDL cholesterol with MMSE scores. **(C)** Correlation of triglycerides with MMSE scores. **(D)** Correlation of total cholesterol with MMSE scores. **(E)** Correlation of cholesterol ratio with MMSE scores.

Correlation analysis was performed in order to observe the association of concentrations of LDL cholesterol, HDL cholesterol, triglycerides, total cholesterol and cholesterol ratio with the extent of CI. Correlation test revealed that the strongest negative correlation was observed with cholesterol ratio (*r* = −0.562; ***p < 0.001) followed by LDL cholesterol (*r* = −0.428; ***p < 0.001), total cholesterol (*r* = −0.39; ***p < 0.001) and triglycerides (*r* = −0.329; ***p < 0.001), respectively (Figures 3A–E). A strong positive correlation was observed with HDL cholesterol concentration (*r* = 0.467; ***p < 0.001) and MMSE score.

### Tau Levels in Serum, ROCs in CI Severity and Its Correlation With MMSE, Lipids Profile, and Metals Levels

**Figure 4:**
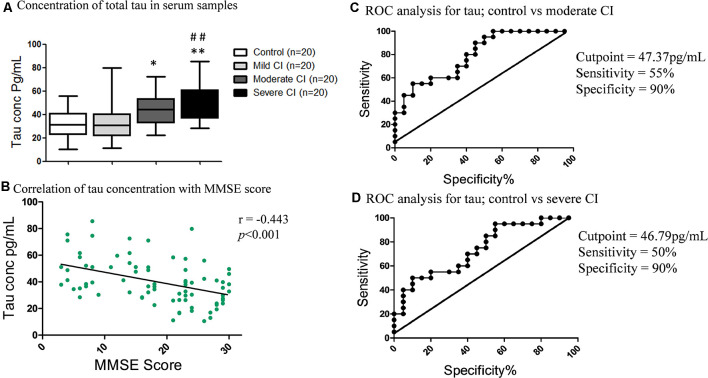
Serum tau levels and its correlation with MMSE and Receiver Operating Characteristic (ROC) curves. **(A)** Concentration of total-tau in serum samples in studied subjects. *p < 0.05, **p < 0.01 compared with age-matched healthy control group; ^##^*p* < 0.01 comparison with mild cognitive impaired group; *n* = sample size. **(B)** Correlation of tau concentration with MMSE scores. ROC analysis of serum tau proteins between **(C)** Control vs. moderate CI and **(D)** Control vs. severe CI groups.

The concentration of serum total-tau levels increased with increasing severity of CI (Figure 4A). The total-tau concentration in subjects with severe CI (50.05 ± 3.68 pg/ml; **p < 0.01) and moderate CI (44.94 ± 3.19 pg/ml; *p < 0.05) were significantly higher than the age-matched healthy control (31.8 ± 2.79 pg/ml). There were significantly low levels of tau in subjects with mild CI (34.4 ± 3.76 pg/ml; **p < 0.01) in comparison to severe CI group. No significant difference was observed among mild CI group and age-matched healthy control (Figure 4A). A significant and downhill linear correlation was found between total-tau levels and MMSE score (*r* = −0.443; ***p < 0.001; Figure 4B).

ROC analysis revealed that tau levels showed the best results in moderate CI followed by severe CI, but not very accurate in mild CI (Supplementary Figure S1). In the case of moderate CI vs. control, the AUC for tau was 0.80 and the cutoff value was 47.37 pg/ml; with a sensitivity of 55% and specificity of 90% to detect moderate CI patients (Figure 4C). While, in case of severe CI vs. control, the AUC was 0.75, the cutoff concentration was 46.79 pg/ml; with a sensitivity of 50% and specificity of 90% to detect severe CI (Figure 4D).

**Figure 5:**
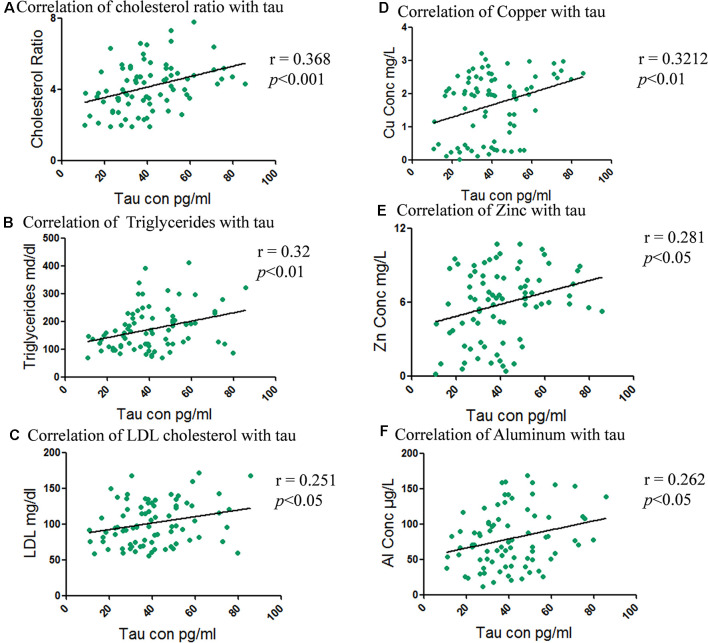
Correlation between total and fractional serum cholesterol levels with total tau concentration and metals levels with tau. **(A)** Correlation of cholesterol ratio with tau. **(B)** Correlation of triglycerides with tau. **(C)** Correlation of LDL cholesterol with tau. **(D)** Correlation of total tau with Copper. **(E)** Correlation of total tau with Zinc. **(F)** Correlation of total tau with Aluminum.

Correlation analysis of tau concentration and cholesterol fractions showed the strongest positive correlation with cholesterol ratio (*r* = 0.368; ***p < 0.001) followed by triglyceride (*r* = 0.32; **p < 0.01) and least with LDL cholesterol (*r* = −0.251; *p < 0.05; Figures 5A–C). A negative correlation between the HDL cholesterol (*r* = −0.245; *p < 0.05) and tau concentration whereas no correlation with total cholesterol was shown (Supplementary Figures S2A,B).

Metal concentrations were correlated with tau levels to determine any association among the elevated levels of metals with the levels of serum tau proteins. Pearson’s correlation demonstrated significant correlation of tau with Cu (*r* = 0.32; **p < 0.01), followed by Zn (*r* = 0.28; *p < 0.05), Al (*r* = 0.26; *p < 0.05) as shown in Figures 5D–F. While Mn (*r* = 0.23; *p < 0.05) was weakly correlated and Pb (*r* = −0.251; ns) and Cd (*r* = −0.251; ns) were non-significant (Supplementary Figures S2C–E).

### Amyloid Beta-42 Levels in Serum, ROCs in CI Severity and Its Correlation With MMSE, Lipids Profile and Metals Levels

**Figure 6:**
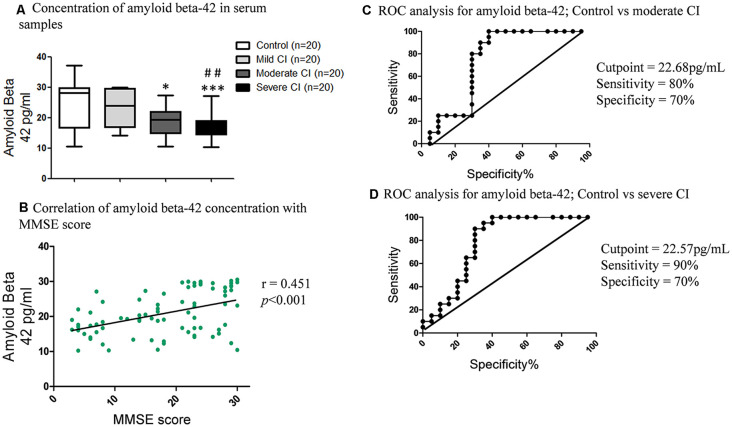
Serum amyloid beta-42 levels and its correlation with MMSE, and ROC curves. **(A)** Concentration of serum amyloid beta-42 in serum samples in studied subjects. *p < 0.05, ***p < 0.001 compared with age-matched healthy control group; ^##^*p* < 0.01 comparison with mild cognitive impaired group; n = sample size. **(B)** Correlation of serum amyloid beta-42 concentration with MMSE scores. ROC curves of **(C)** Control vs. moderate CI and **(D)** Control vs. severe CI.

The amyloid beta-42 levels declined with the increase in CI (Figure 6A). The amyloid beta-42 concentration was low in subjects diagnosed with severe CI (17.05 ± 1.68 pg/ml) compared to mild CI (23.52 ± 1.36; **p < 0.01 pg/ml) and age matched healthy controls (24.68 ± 1.68 pg/ml; ***p < 0.001). Correlation analyses revealed significant positive correlation of amyloid beta-42 levels and MMSE score (*r* = 0.451; ***p < 0.001; Figure 6B).

ROC analyses revealed that amyloid beta-42 did now show promising results for mild CI vs. control (Supplementary Figure S3). In case of moderate CI vs. control, the AUC was 0.74, the cutoff value was 22.68 pg/ml; with sensitivity 80% and specificity 70% (Figure 6C). While in case of severe CI vs. control, the AUC was 0.79, the cutoff value of amyloid beta-42 was 22.57 pg/ml; with a sensitivity of 90% and specificity of 70% to detect severe cognitively impaired patients (Figure 6D).

**Figure 7:**
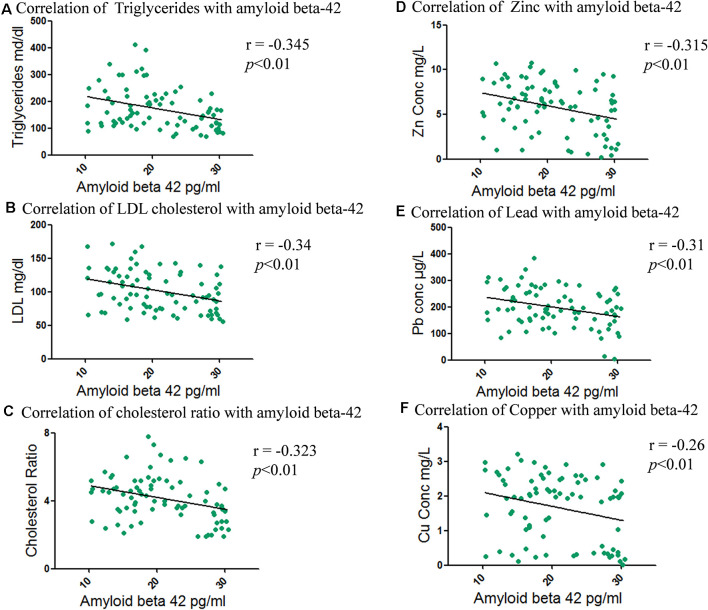
Correlation between total and fractional serum cholesterol levels with amyloid beta-42 and metals levels with amyloid beta-42. **(A)** Correlation of triglycerides with amyloid beta-42. **(B)** Correlation of LDL cholesterol with amyloid beta-42. **(C)** Correlation of cholesterol ratio with amyloid beta-42. **(D)** Correlation of amyloid beta-42 with Zinc. **(E)** Correlation of amyloid beta-42 with Lead. **(F)** Correlation of amyloid beta-42 with Copper.

Furthermore amyloid beta-42 correlation with cholesterol fractions was evaluated and a negative correlation was seen in the order of triglyceride (*r* = −0.345; **p < 0.01), LDL cholesterol (*r* = −0.34; **p < 0.01) and cholesterol ratio (*r* = −0.323; **p < 0.01; Figures 7A–C). A positive correlation with HDL cholesterol (*r* = 0.292; **p < 0.01) and amyloid beta-42 concentration, whereas weak negative correlation of total cholesterol (*r* = −0.28; *p < 0.05) with amyloid beta-42 was seen (Supplementary Figures S4A,B).

When amyloid beta-42 concentration was correlated with metals, it was found that amyloid beta-42 is negatively correlated with the concentration of Zn (*r* = −0.32; **p < 0.01), followed by Pb (*r* = −0.31; **p < 0.01) and Cu (*r* = −0.26; **p < 0.01) as shown in Figures 7D–F. Al was marginally negatively associated (*r* = −0.25; *p < 0.05) whereas Mn (*r* = −0.21; ns) and Cd (*r* = −0.20; ns) showed non-significant correlation (Supplementary Figures S4C–E).

### ROC Analysis of Metals to Detect Mild CI, Moderate CI and Severe CI

**Figure 8:**
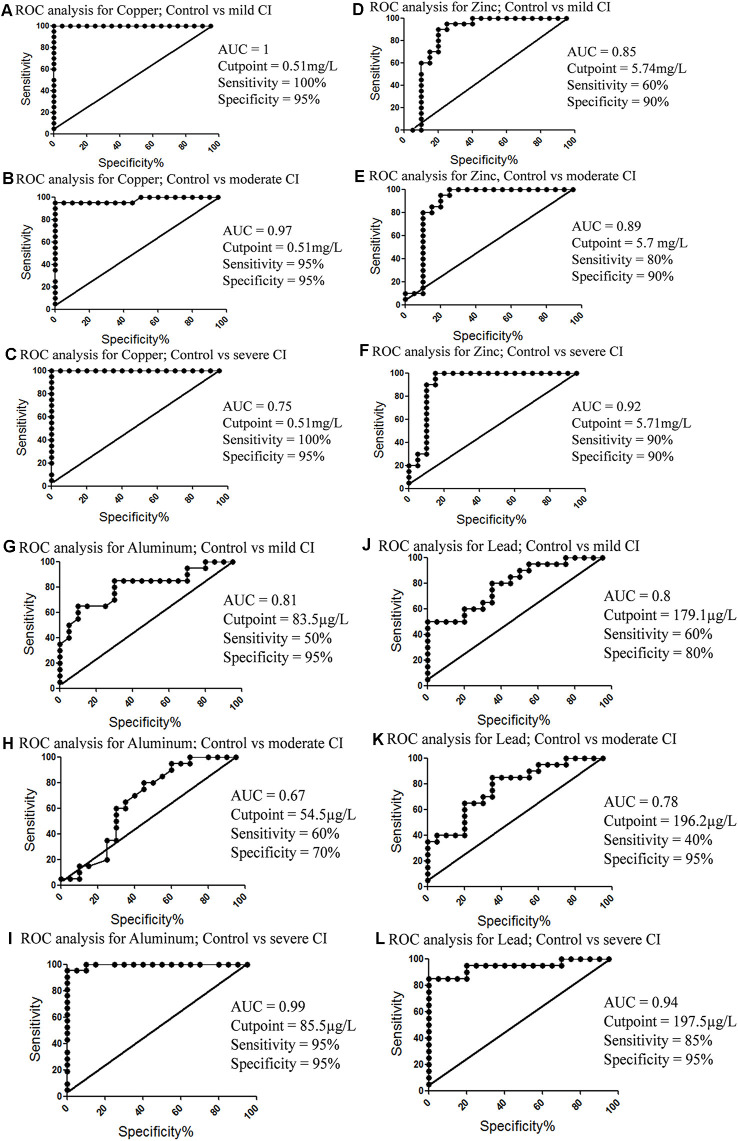
ROC analysis of metals concentrations in different groups. **(A)** Copper concentration; control vs. mild CI. **(B)** Copper concentration; control vs. moderate CI. **(C)** Copper concentration; control vs. severe CI. **(D)** Zinc concentration; control vs. mild CI. **(E)** Zinc concentration; control vs. moderate CI. **(F)** Zinc concentration; control vs. severe CI. **(G)** Aluminum concentration; control vs. mild CI. **(H)** Aluminum concentration; control vs. moderate CI. **(I)** Aluminum concentration; control vs. severe CI. **(J)** Lead concentration; control vs. moderate CI. **(K)** Lead concentration; control vs. severe CI. **(L)** Lead concentration; control vs. mild CI.

Metals levels were estimated in these subjects (Iqbal et al., 2018) and their ROC analyses for diagnostic value were evaluated here. It was found that Cu showed the best diagnostic value (Figures 8A–C), followed by Zn (Figures 8D–F), Al (Figures 8G–I), and Pb (Figures 8J–L). ROC analyses of Mn and Cd were also analyzed (Supplementary Figures S5A–F).

Finally, we added amyloid beta-42 and total tau concentration and ROC analysis was performed. The results did not show very promising ROC values of these combined biomarkers (Supplementary Figure S6).

## Discussion

The decline in both physical and cognitive function has been associated with increased aging. A physiological link exists between physical frailty and cognitive decline. These underlying processes include chronic inflammation, impaired hypothalamic-pituitary axis stress response, imbalanced energy metabolism, mitochondrial dysfunction, oxidative stress, and neuroendocrine dysfunction (Freitas et al., 2019; Adams et al., 2019; Yang et al., 2019; Ma and Chan, 2020). Blood-based biomarkers for CI are very important and can be effectively used to promote healthy brain aging, for screening and diagnosis at every stage of the dementias, assessment of risk to the disease, and may also be helpful in drug discovery approaches. In many cases, blood-based biomarkers lack specificity and sensitivity; hence their clinical application is limited. Tau proteins that are associated with axonal damage and amyloid proteins linked with plaque formation have been used in clinical trials as CSF based diagnostic markers for certain neurological diseases (Cummings, 2011). Interestingly, the present study showed that the concentration of both serum tau and amyloid beta-42 were significantly different in cases of moderate and severe CI compared to mild CI and age-matched healthy controls. These findings add further evidence that serum amyloid and tau levels may improve prediction of CI, are associated with various manifestations of CI, and may represent a useful biomarker for evaluating at-risk individuals in CI prevention trials.

A recent study reported that high levels of plasma tau was associated with a decrease in logical memory, volume of gray matter, and hippocampus (Chiu et al., 2014). Our study also showed similar results that patients of severe CI have increased levels of serum tau and tau protein levels are negatively correlated with the MMSE score. The ROC analysis determined the cutoff concentrations which were highly sensitive and specific to differentiate mild, moderate, and severe CI from age-matched healthy controls. The cutoff values from ROC analysis have depicted that tau concentration can be efficiently used to detect severe CI. Metals contribute to the pathology of CI by aggregating amyloid-beta and tau phosphorylation (Kim et al., 2018). The hyper-phosphorylation of tau leads to oxidative stress (Mandelkow and Mandelkow, 1998). Cu and Zn add to the tau pathology by directly binding with tau proteins (Ma et al., 2006; Huang et al., 2014) while Pb and Al contribute to apoptosis (Zhang et al., 2012; Brown et al., 2019). Our results show that tau concentration and metals are directly correlated, where increasing metals concentration was directly affecting tau increases, and this was found in the order of Cu, Zn, and Al, suggesting the modulatory roles of these metals.

Amyloid-beta levels are in dynamic equilibrium at the peripheral and cerebral level (Wang et al., 2006). The deposition of amyloid beta-42 in the brain might reduce plasma levels of amyloid beta-42 (Iadecola, 2003). The accumulation of extracellular amyloid beta-42 can induce the formation of intracellular NFTs that causes organelle stress leading to neurodegeneration and CI in AD. This phenomenon is termed the “snowball hypothesis” (Bi et al., 2019). The beta and gamma secretases enzyme that is involved in the amyloid beta-42 formation is dominantly localized in cholesterol-rich domains of the plasma membrane (Ehehalt et al., 2003). Studies have also shown that the cellular concentration of cholesterol might regulate the concentration and production of amyloid-beta peptides. Increased levels of cellular cholesterol shift the metabolism towards the amyloidogenic pathway however decreased cholesterol results in the non-amyloidogenic pathway (Bodovitz and Klein, 1996; Kojro et al., 2001). Different lipid-lowering therapies have been shown to interfere indirectly with amyloid-beta protofibrils by either cholesterol-dependent or cholesterol independent pathways (Shakour et al., 2019). Lipid-lowering therapy has been shown to ameliorate asymptomatic intracranial atherosclerosis, which is a risk factor for vascular CI and dementia (Xie et al., 2018; Zou et al., 2018; Miao et al., 2019; Shetty et al., 2019). Atorvastatin treatment also improved cognitive outcomes and induced anti-inflammatory response in a rat model for a chronic subdural hematoma and intracerebral hemorrhage (Quan et al., 2019).

In the current study, we reported that the levels of serum amyloid-beta decreased with the progression of the disease from mild to severe. There was a positive correlation observed between levels of amyloid beta-42 and the MMSE score. The ROC cutoff value revealed that amyloid beta-42 is more sensitive and specific to determine the different stages of disease compared to tau. The high sensitivity and specificity of serum tau and amyloid beta-42 might be useful to diagnose the CI with high accuracy. Al, Cu, and Zn were found in large quantities in aggregates of amyloid beta-42 (Mantyh et al., 1993) and were strongly associated with amyloid beta-42 levels in our study. Cu is required for normal brain function and Cu metabolism is dysregulated in brain aging (Braidy et al., 2017). Alterations in copper fluxes have also been reported in murine brain aging using ^64^CuCl_2_ as a radiotracer (^64^CuCl_2_-PET/CT; Peng et al., 2018). Metals, including Cu and redox metals, are directly involved in the generation of amyloid plaques and indirectly by inducing oxidative stress/damage (Smith et al., 2006; Liguori et al., 2018). High Mn concentration also induces the amyloid-beta related cognitive decline in previous studies (Tong et al., 2014). Our study revealed a similar finding that metals increased the amyloid-beta aggregates and ultimately leading to decreed amyloid-beta levels in blood serum.

This study also explored the association between adverse lipid profiles and CI. We found that a low serum concentration of HDL cholesterol was linked to CI. Serum concentrations of total cholesterol, triglycerides, and LDL cholesterol showed association with CI therefore we can conclude that alteration in cholesterol metabolism in the brain might contribute to the pathology of CI. The subjects included in this study did not have cardiovascular disease or hypertension. The association of these parameters was not confounded by living standards or education status.

An association between high levels of total cholesterol, LDL cholesterol, triglycerides; and a low concentration of HDL cholesterol are risk factors for cardiovascular disease that have been previously documented (Sacco et al., 2001). Lipoprotein-associated phospholipase A2 and superoxide dismutase are linked to regulating neuroinflammation (Zhu et al., 2019). Lipoprotein cholesterol and high sensitivity C-reactive protein have also been shown to correlate with Parkinson’s disease severity (Yang et al., 2020). LDL cholesterol and plasma cystatin—a protein produced by nucleated cells—has been shown to differentiate progressive supranuclear palsy from healthy subjects and predict disease severity (Weng et al., 2018). Our study reported similar results with the risk of CI and its propagation. In this study, HDL cholesterol was highly correlated with the CI as compared to LDL cholesterol, triglycerides, and total cholesterol. HDL cholesterol is the predominant lipoprotein in the human brain (Olesen and Dagø, 2000) and prevents aggregation of amyloid proteins (Koudinov et al., 1998), and may prevent the development and progression of the disease. The association between low levels of HDL cholesterol and progression of CI was irrespective of the presence of stroke, hypertension, and cardiovascular disease. HDL cholesterol has anti-inflammatory properties (Cockerill et al., 1995), and inflammation is considered to play a contributory if not causal role in neurodegenerative processes (Grundke-Iqbal et al., 1986; McGeer and McGeer, 1998).

Our study has also shown that individuals with high concentrations of LDL cholesterol were diagnosed with severe CI. The stronger correlation was demonstrated with LDL cholesterol followed by HDL cholesterol. Previous studies have also reported that individuals with AD have significantly higher LDL cholesterol and lower HDL cholesterol hence influencing AD pathology (Kuo et al., 1998; Moroney et al., 1999). High levels of LDL cholesterol and total cholesterol leads to microglial activation and amyloid-beta formation and may be directly involved in the pathobiology of dementia (Streit and Sparks, 1997). High serum total cholesterol may be a risk factor for CI and its progression. Total cholesterol was positively correlated with the MMSE score. One study revealed that total cholesterol is an independent risk factor for dementia and AD (Notkola et al., 1998). As well, Anstey et al showed an association between high midlife total cholesterol and cognitive decline (Anstey et al., 2008).

Triglycerides mediate CI, possibly by impairing maintenance of the *N*-methyl-D-aspartate component of hippocampal long-term potentiation and increasing oxidative stress (Razay et al., 2007; Farr et al., 2008). Our study is also consistent with previous findings, since serum triglyceride levels increased, in line with increased CI. There was a negative correlation observed between triglycerides and MMSE scores. Hence lipid-lowering therapy can improve neurological outcome (Quan et al., 2019).

This study provides insights on aging and mechanisms of CI as these are critical for novel therapies that might prevent or cure multiple age-related diseases. Among metals, Cu and Al were found to be significantly correlated with amyloid and tau. Whereas ROC analysis has also shown that Cu, Zn, and Al levels can be used as diagnostic markers for CI. Blood-based amyloid beta-42 and tau proteins might be used as specific biomarkers to evaluate the extent of cognitive deficits. Whereas, lipid and metal dyshomeostasis may contribute to the pathology of CI and its progression.

## Conclusion

In conclusion, the low serum concentration of HDL cholesterol, high LDL cholesterol, total cholesterol, and triglycerides was associated with the progression of CI and clinical diagnosis of CI. The serum proteins, total-tau, and amyloid beta-42 may be practical to diagnose CI with high sensitivity and specificity. These findings are of clinical importance because they suggest that increasing HDL and lowering LDL cholesterol, total cholesterol, triglycerides, and metals might prevent the development and progression of CI and quantifying total-tau, Cu, Zn, Al and amyloid beta-42 may collectively represent a useful diagnostic tool.

## Data Availability Statement

All datasets generated for this study are included in the article/Supplementary Material.

## Ethics Statement

The studies involving human participants were reviewed and approved by Internal Review Board (IRB) ASAB NUST. The patients/participants provided their written informed consent to participate in this study.

## Author Contributions

TA: project supervisor. TA, NB, and GI: study design. GI: data collection. GI: laboratory work/experimental. GI and TA: data analysis. TA, NB, and GI: manuscript preparation.

## Conflict of Interest

The authors declare that the research was conducted in the absence of any commercial or financial relationships that could be construed as a potential conflict of interest.
