# Supplementary material for: Blood-Based Biomarkers for Predictive Diagnosis of Cognitive Impairment in a Pakistani Population
Source: Front Aging Neurosci. 2020 Jul 22;12:223. doi: 10.3389/fnagi.2020.00223 (PMC7396488; doi:10.3389/fnagi.2020.00223)
Supplement: Supplementary file 1 [file Date_Sheet_1.PDF]

Blood-based biomarkers for predictive diagnosis of cognitive impairment in a Pakistani population

Ghazala Iqbal[a], Nady Braidy[b], Touqeer Ahmed[a]*

[a]Neurobiology Laboratory, Department of Healthcare Biotechnology, Atta-ur-Rahman School of Applied Biosciences, National University of Sciences and Technology, Sector H-12, Islamabad – 44000, Pakistan

[b]Centre for Healthy Ageing, School of Psychiatry, Faculty of Medicine, University of New South Wales, Sydney, Australia

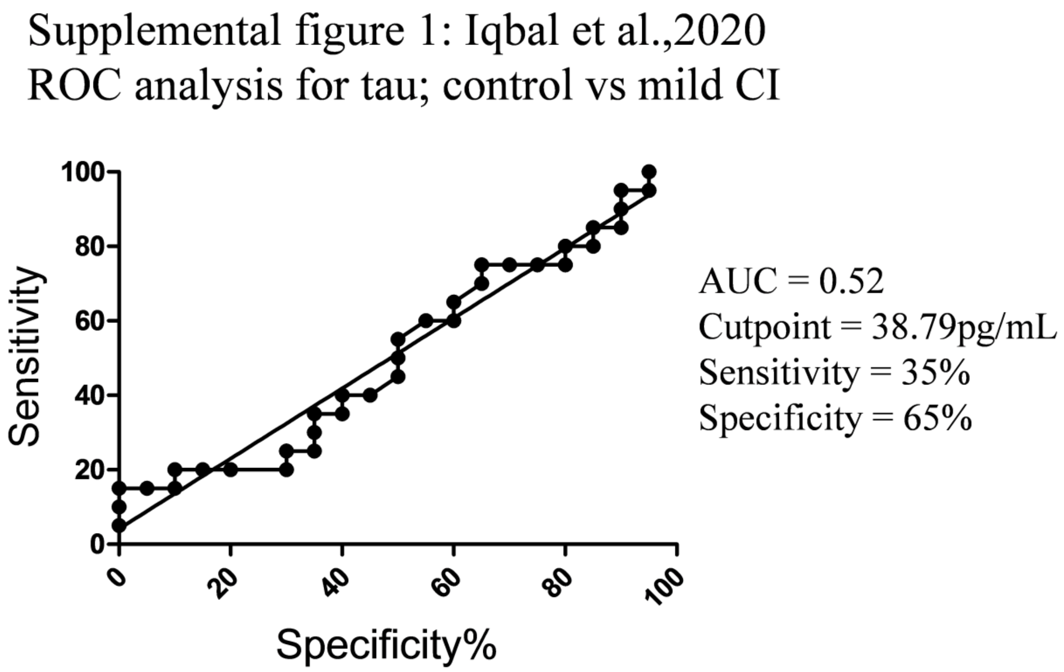

Supplemental figure 1: Receiver Operating Characteristic (ROC) analysis of serum tau proteins between Control vs. mild CI groups.

Supplemental figure 2: Iqbal et al.,2020

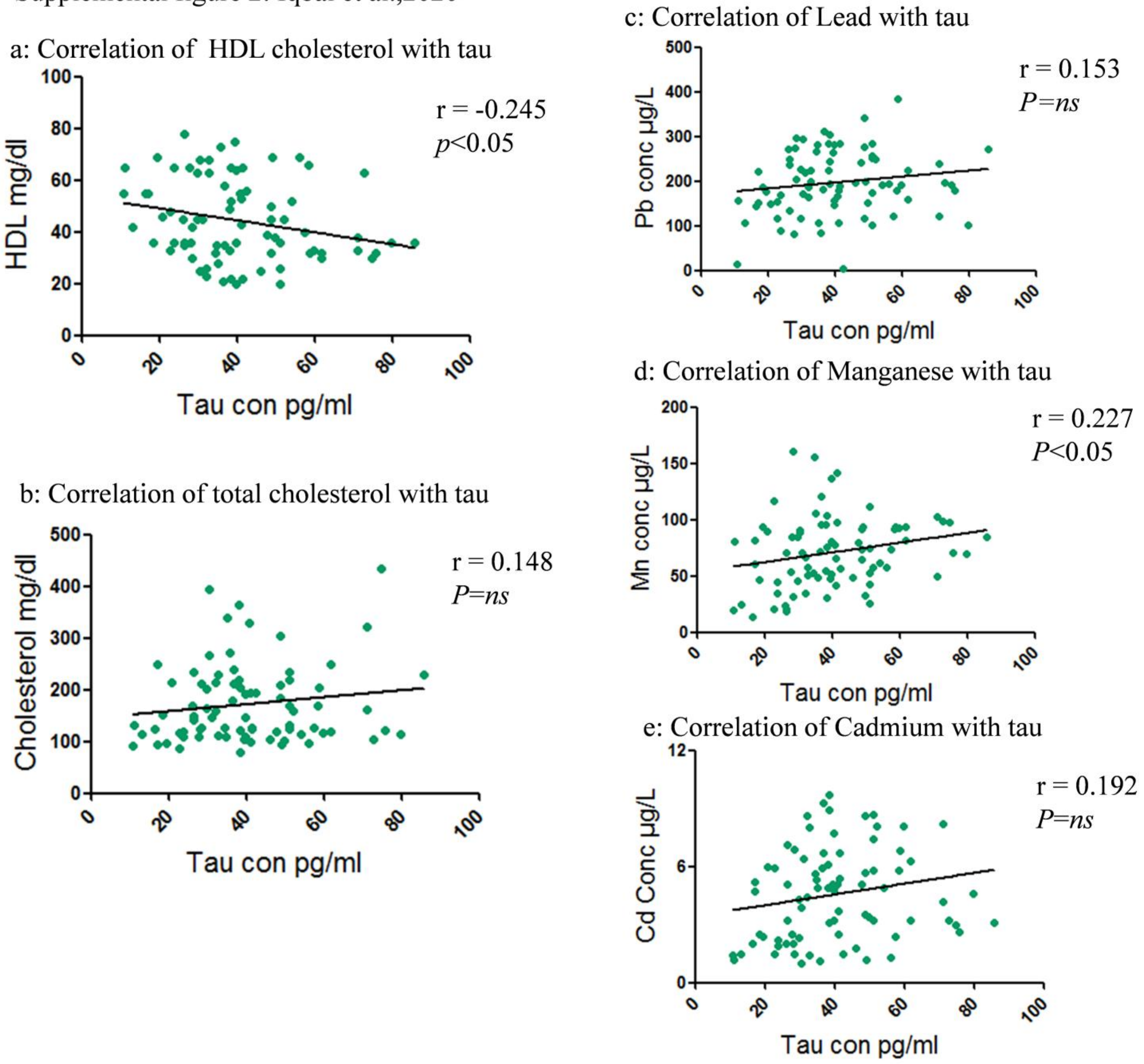

Supplemental figure 2: (a) Correlation of HDL cholesterol with tau. (b) Correlation of total cholesterol with tau. (c) Correlation of total tau with Lead. (d) Correlation of total tau with Manganese. (e) Correlation of total tau with Cadmium.

Supplemental figure 3: Iqbal et al.,2020

ROC analysis for amyloid beta-42; Control vs mild CI

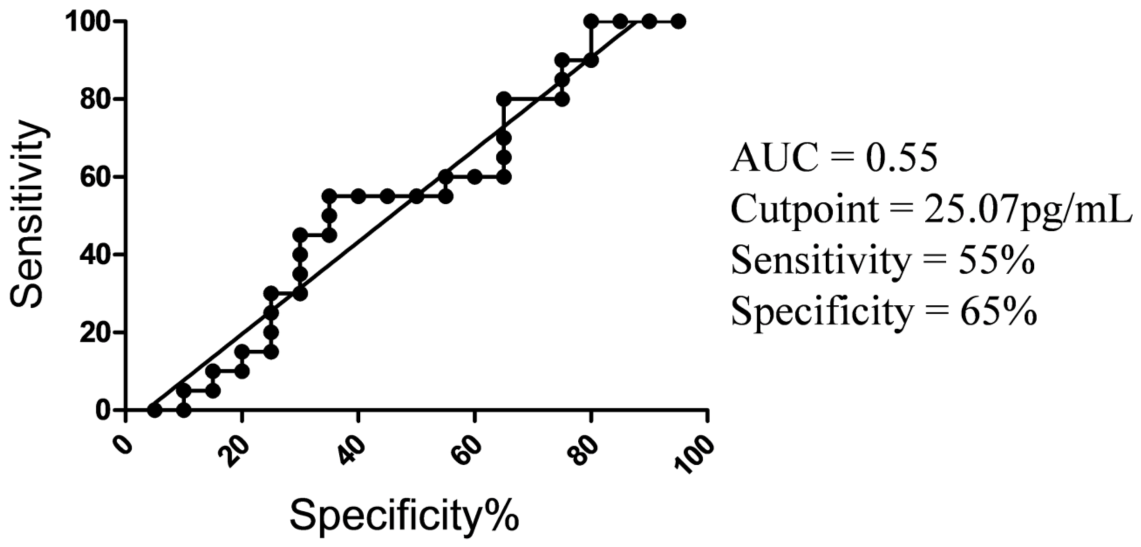

Supplemental figure 3: Receiver Operating Characteristic (ROC) analysis of serum amyloid beta-42 proteins in Control vs. mild CI.

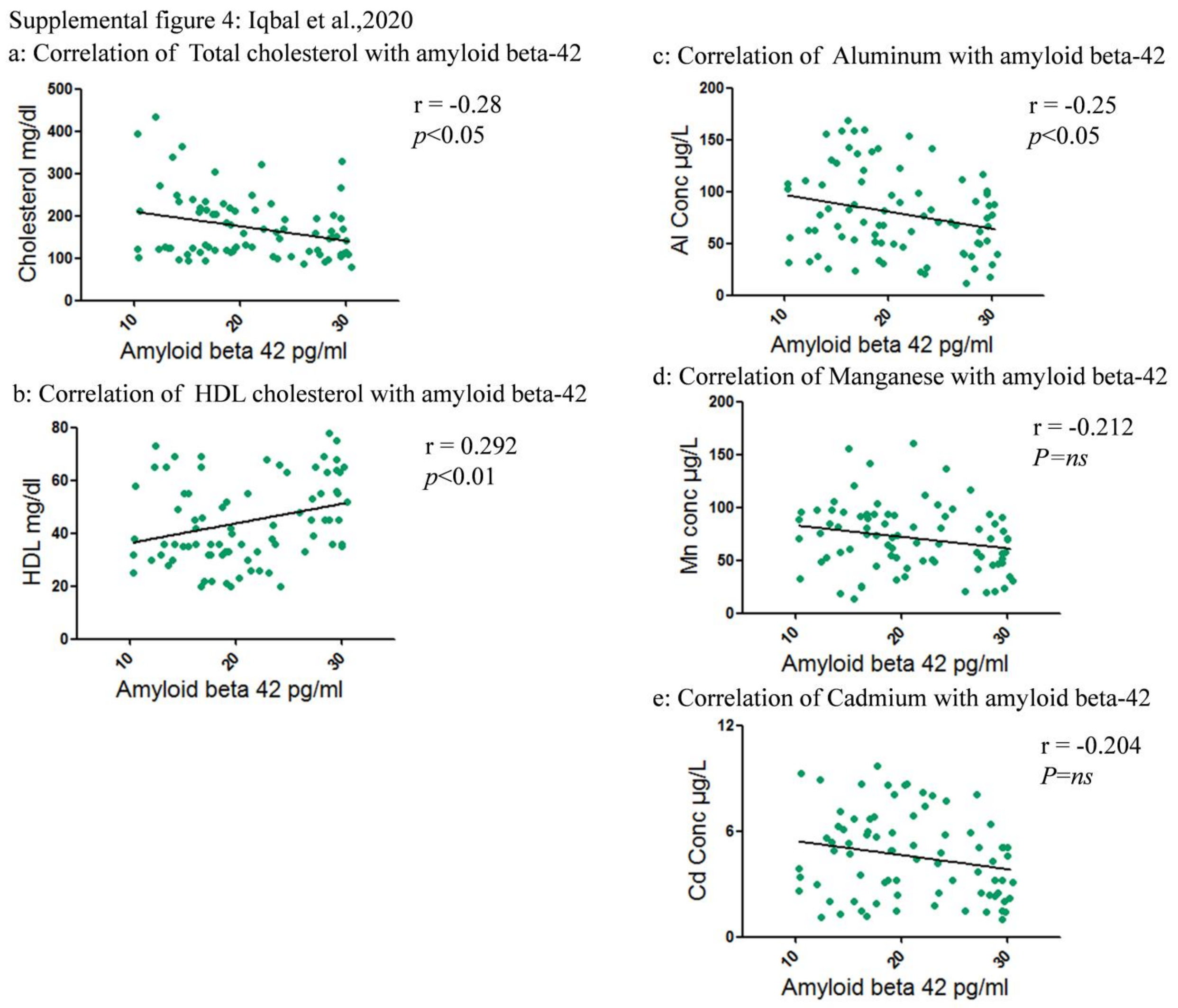

Supplemental figure 4: (a) Correlation of total cholesterol with amyloid beta-42. (b) Correlation of HDL cholesterol with amyloid beta-42. (c) Correlation of amyloid beta-42 with Aluminum. (d) Correlation of amyloid beta-42 with manganese. (e) Correlation of amyloid beta-42 with Cadmium.

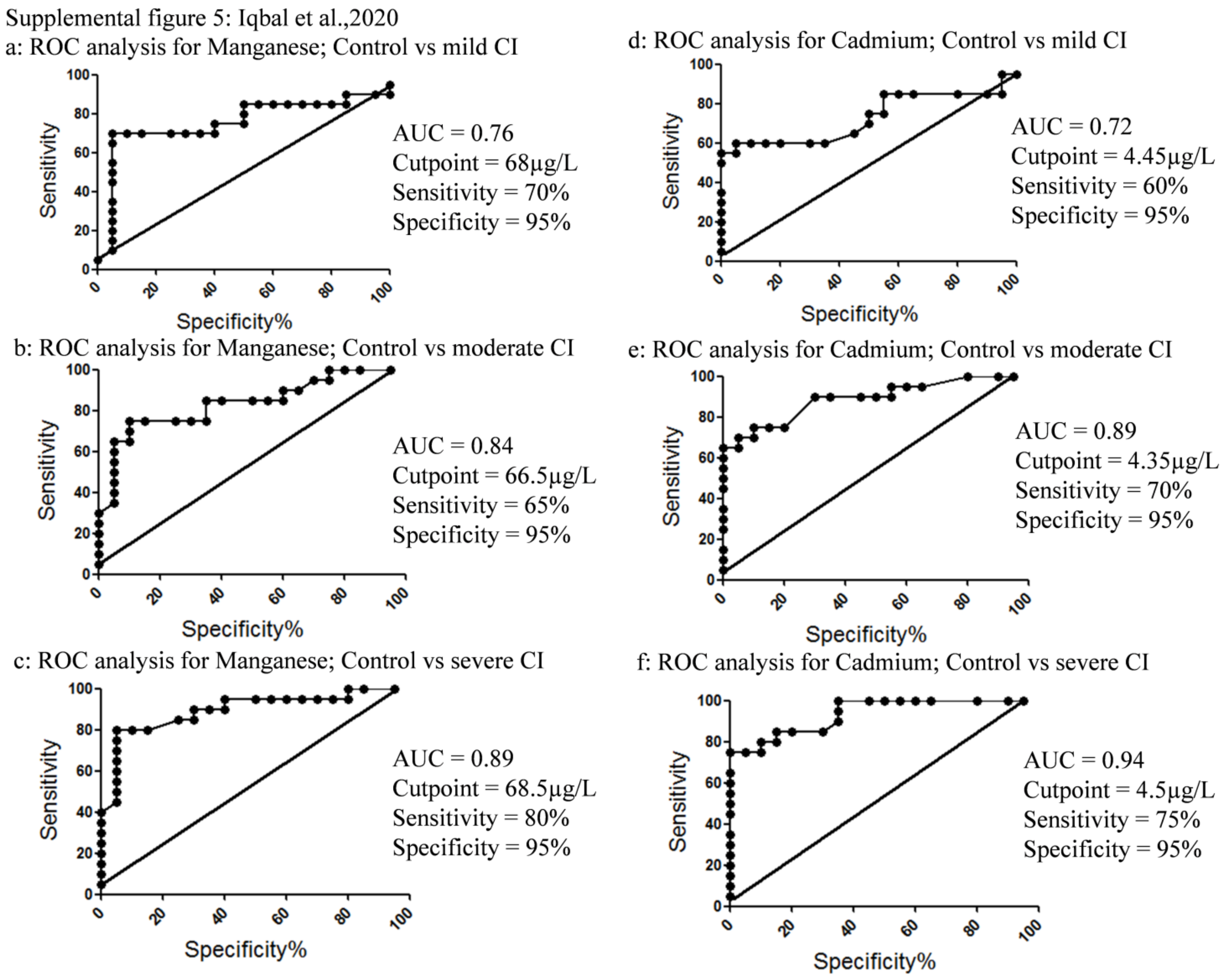

Supplemental figure 5: (a) Manganese concentration; control vs. mild CI. (b) Manganese concentration; control vs. moderate CI. (c) Manganese concentration; control vs. severe CI. (d) Cadmium concentration; control vs. mild CI. (e) Cadmium concentration; control vs. moderate CI. (f) Cadmium concentration; control vs. severe CI.

Supplemental figure 6: Iqbal et al.,2020

a: ROC analysis for amyloid beta-42+ tau; Control vs mild CI

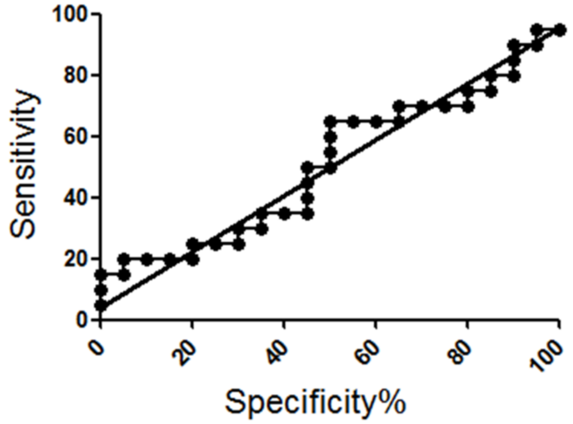

AUC = 0.51
Cutpoint = 70.24pg/mL
Sensitivity = 20%
Specificity = 95%

b: ROC analysis for amyloid beta-42+ tau, Control vs moderate CI

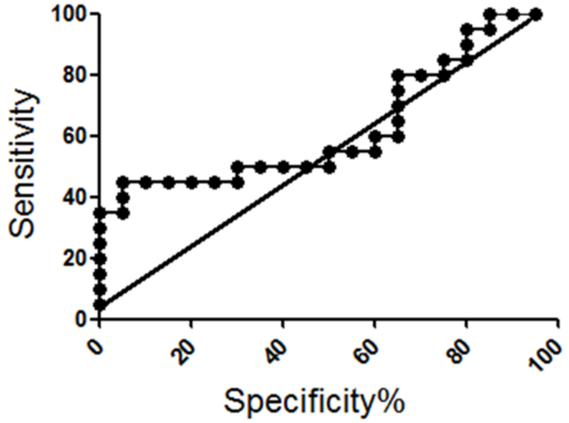

AUC = 0.63
Cutpoint = 70.4 pg/mL
Sensitivity = 45%
Specificity = 95%

c: ROC analysis for amyloid beta-42+ tau; Control vs severe CI

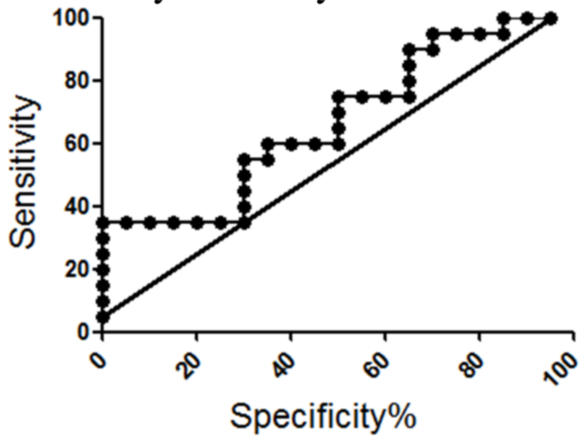

AUC =0.67
Cutpoint = 71pg/mL
Sensitivity = 35%
Specificity = 95%

Supplemental figure 6: Receiver Operating Characteristic (ROC) analysis of serum amyloid beta-42+total tau proteins among the groups. (a) Control vs. mild CI. (b) Control vs. moderate CI. (c) Control vs. severe CI.
